# YOLO-WL: A Lightweight and Efficient Framework for UAV-Based Wildlife Detection

**DOI:** 10.3390/s26030790

**Published:** 2026-01-24

**Authors:** Chang Liu, Peng Wang, Yunping Gong, Anyu Cheng

**Affiliations:** 1 Intelligent Manufacturing and Automobile School, Chongqing Polytechnic University of Electronic Technology, Chongqing 401331, China; 17612323143@163.com (C.L.); gongyunping@bupt.cn (Y.G.); 2 School of Automation, Chongqing University of Posts and Telecommunications, Chongqing 400065, China; wstchhwp@126.com

**Keywords:** UAV, wildlife detection, small object detection, feature fusion

## Abstract

Accurate wildlife detection in Unmanned Aerial Vehicle (UAV)-captured imagery is crucial for biodiversity conservation, yet it remains challenging due to the visual similarity of species, environmental disturbances, and the small size of target animals. To address these challenges, this paper introduces YOLO-WL, a wildlife detection algorithm specifically designed for UAV-based monitoring. First, a Multi-Scale Dilated Depthwise Separable Convolution (MSDDSC) module, integrated with the C2f-MSDDSC structure, expands the receptive field and enriches semantic representation, enabling reliable discrimination of species with similar appearances. Next, a Multi-Scale Large Kernel Spatial Attention (MLKSA) mechanism adaptively highlights salient animal regions across different spatial scales while suppressing interference from vegetation, terrain, and lighting variations. Finally, a Shallow-Spatial Alignment Path Aggregation Network (SSA-PAN), combined with a Spatial Guidance Fusion (SGF) module, ensures precise alignment and effective fusion of multi-scale shallow features, thereby improving detection accuracy for small and low-resolution targets. Experimental results on the WAID dataset demonstrate that YOLO-WL outperforms existing state-of-the-art (SOTA) methods, achieving 94.2% mAP@0.5 and 58.0% mAP@0.5:0.95. Furthermore, evaluations on the Aerial Sheep and AI-TOD datasets confirm YOLO-WL’s robustness and generalization ability across diverse ecological environments. These findings highlight YOLO-WL as an effective tool for enhancing UAV-based wildlife monitoring and supporting ecological conservation practices.

## 1. Introduction

Wildlife detection involves identifying animal species in natural habitats through direct observation or modern technologies such as computer vision, AI, and DNA analysis. As global biodiversity faces growing threats from habitat loss, climate change, and illegal poaching, accurate detection has become crucial for protecting endangered species and maintaining ecosystem stability [1,2]. It provides essential data, such as population size, distribution, and behavior, needed to assess ecological health and develop science-based conservation strategies.

In recent years, unmanned aerial vehicles (UAVs) have become valuable tools for wildlife monitoring and conservation, offering high efficiency, safety, and wide-area coverage. Unlike traditional manual surveys, UAVs can rapidly monitor complex terrains—such as forests, wetlands, and mountains—equipped with sensors like high-definition cameras, thermal imaging, and multispectral detectors. These enable remote, non-invasive, real-time data collection with minimal disturbance to animals. Integrated with AI-based image recognition, UAVs can automatically detect species in aerial imagery, significantly improving monitoring accuracy and efficiency. Their all-weather and low-light operation capabilities further support long-term data acquisition, enabling comprehensive ecological monitoring. This continuous data flow is essential for studying population dynamics, migration, and ecosystem responses, providing strong scientific support for conservation policies [3,4,5].

The rapid advancement of deep learning has revolutionized wildlife identification from UAV-captured imagery through Convolutional Neural Networks (CNNs) [6,7,8]. Thanks to their powerful feature extraction and pattern recognition capabilities, these methods can achieve high-accuracy classification of various species, even under complex and dynamic natural backgrounds. Numerous researchers have conducted extensive studies on wildlife identification in aerial imagery. For example, Roy et al. [9] introduced WilDect-YOLO, a high-performance object detection framework designed to enable real-time monitoring of endangered wildlife species. A YOLO-SAG-based method was later proposed by Chen et al. [10], which offers improved trade-offs between detection accuracy and computational efficiency. He et al. [11] proposed ALSS-YOLO, a compact and efficient detector specifically designed for wildlife detection in thermal infrared (TIR) aerial images. Ma et al. [12] proposed an improved version of YOLOv5s for lightweight wildlife detection in complex environments. Zhang et al. [13] developed a Channel-Enhanced RetinaNet (CE-RetinaNet), achieving high-precision detection in infrared imagery. Ye et al. [14] presented an improved ADD-YOLO algorithm tailored to enhance animal detection performance in aerial images. Furthermore, Jia et al. [15] proposed WL-YOLO, a lightweight detection algorithm that demonstrates superior performance in handling occlusion and complex environmental conditions.

Nevertheless, most existing approaches address only one or two aspects of the problem in isolation. For instance, some methods enhance small-object detection but neglect fine-grained discrimination among visually similar species, while others improve robustness to occlusion or background clutter yet remain vulnerable to the severe information loss caused by low-resolution imaging. Crucially, the lack of a unified design that simultaneously tackles weak inter-class discriminability, extreme scale variation, and complex environmental interference limits the practical applicability of current solutions in real-world ecological monitoring scenarios.

However, wildlife identification from UAV imagery still faces significant technical and environmental challenges. Many species exhibit similar morphological features—especially closely related or juvenile individuals—making visual differentiation difficult. High-altitude perspectives further limit the visibility of behavioral cues like posture and movement, reducing the effectiveness of behavior-assisted identification. Dense vegetation and complex terrain often cause partial or full occlusion of animals, particularly in forested or mountainous areas. Lighting variations due to shadows and solar angles lead to inconsistent image quality, hindering stable feature extraction. Similarities in color and texture between animals and background elements (e.g., rocks, dry leaves) increase false positive rates. Moreover, animals typically appear as small, low-resolution targets due to high-altitude UAV flight, limiting pixel-level detail and posing major challenges for vision-based algorithms reliant on high-quality input.

This paper proposes a UAV-based wildlife identification algorithm built on YOLO-WL, specifically optimized to address challenges of visually similar species, environmental disturbances, and small target sizes. The main contributions are:

1. To address the challenge of visually similar wildlife species that are difficult to distinguish due to high inter-class similarity, we propose the MSDDSC module, which integrates multi-scale structures with dilated depthwise separable convolution to effectively fuse local details and global context, thereby expanding the receptive field and enhancing discriminative capability for morphologically similar animals.
2. To tackle complex environmental disturbances such as occlusion from dense vegetation, terrain variations, and illumination changes that introduce strong background noise, we design the MLKSA mechanism, which combines multi-scale feature extraction, large-kernel convolution, and spatial attention to dynamically focus on critical regions across scales and effectively suppress environmental interference.
3. To overcome the limited spatial perception caused by extremely small and low-resolution animal targets in high-altitude UAV imagery, we develop the SSA-PAN network, which incorporates a spatial-guided fusion module into the shallow feature pyramid to enable precise alignment and complementary fusion of shallow multi-scale features, significantly improving detection accuracy for small wildlife targets.

## 2. Related Work

### 2.1. Contextual Information Modeling

Wildlife species often inhabit complex, dynamic environments where challenges such as occlusion, scale variation, uneven lighting, and background clutter degrade visual features and object contours. This undermines local-feature-based detection methods, leading to high false positive and missed detection rates. To overcome these limitations, context-aware modeling has become a key strategy—leveraging semantic information from the surrounding environment (e.g., vegetation, terrain, co-occurring objects) to enhance recognition by jointly understanding the target and its contextual cues.

In context-aware modeling, model design and optimization are typically approached from multiple key perspectives to enhance perception and understanding of complex natural scenes. First, to expand the receptive field, large convolutional kernels [16,17,18] or dilated convolutions [19,20] are employed to alleviate the restricted spatial scope of standard convolutions, thereby capturing richer long-range contextual and semantic dependencies. Second, multi-scale modeling addresses significant scale variations in UAV imagery—caused by differences in distance or posture—through strategies such as multi-branch convolutions [21,22,23], feature pyramid structures, or Atrous Spatial Pyramid Pooling (ASPP) [24,25], allowing the model to capture features across multiple scales and improve discriminative robustness. Third, cross-level semantic integration emphasizes the complementary fusion of low-level and high-level features, where shallow layers provide rich spatial details and deep layers offer high-level semantics. By constructing fusion architectures such as those based on Feature Pyramid Networks (FPN) [26,27,28,29,30], features at different levels are effectively combined to generate more expressive and robust contextual representations. Together, these three aspects—receptive field expansion, scale adaptability, and feature-level fusion—collectively advance contextual information modeling and provide critical support for improving visual recognition performance in challenging environments.

Context-aware modeling is essential for UAV-based wildlife recognition. By integrating receptive field expansion, multi-scale feature learning, and cross-level fusion, the model gains stronger capabilities in handling objects across diverse scales, achieves higher feature representation quality and detection accuracy, and demonstrates improved adaptability and robustness in complex natural environments—thereby offering more reliable support for detection and recognition.

### 2.2. Visual Attention Mechanism

In UAV-based wildlife recognition, animals are often obscured by terrain, illumination changes, or background similarity, making them visually indistinguishable and hard to identify. Visual attention mechanisms address this by dynamically focusing on salient regions of the image.

Visual attention mechanisms enable models to automatically focus on key image regions, selectively enhancing discriminative features while suppressing background noise and irrelevant interference, thereby improving robustness and noise resistance in complex scenarios, especially under occlusion [31,32]. The generated attention weight maps can also be visualized to offer an interpretable explanation of how the model makes its predictions [33,34]. Depending on their design focus, attention mechanisms are typically classified into four types: channel attention, spatial attention, hybrid attention, and self-attention, all of which have demonstrated strong performance across a variety of visual recognition tasks.

Channel Attention adaptively reweights feature channels to emphasize discriminative and informative ones, significantly enhancing feature representational capacity. Representative methods include SENet [35], SENetv2 [36], ECA [37], and EncNet [38]. In contrast, Spatial Attention focuses on the spatial dimension by highlighting salient regions and suppressing background noise, improving perception of target details and context. Notable examples are STN [39] and DCN [40]. Hybrid Attention combines both channel and spatial modeling, enabling comprehensive feature capture from multiple dimensions and improving recognition in complex scenes, with representative approaches such as CBAM [41], BAM [42], and CA [43]. Furthermore, Self-Attention captures long-range pixel dependencies to better model global semantic structures, proving particularly effective for targets with complex spatial distributions. When integrated with Multi-Head Attention, it enables the model to learn diverse representations from multiple subspaces, enhancing expressive power and robustness. Key architectures leveraging this include ViT [44], Swin Transformer [45], DETR [46], Deformable DETR [47], DINO [48], and RT-DETR [49].

Although existing visual attention mechanisms improve feature representation and accuracy, they often neglect multi-scale feature fusion and large receptive field modeling. This leads to imprecise attention localization in complex scenes, increasing susceptibility to background interference and reducing recognition robustness. Additionally, some methods introduce high parameter overhead or over-attend to non-critical regions, harming computational efficiency and discriminative power. To address these issues, there is a need for attention mechanisms that effectively integrate multi-scale features while capturing long-range contextual dependencies.

### 2.3. Comparison with Existing Methods

Despite these advances in contextual modeling and attention mechanisms, most existing detectors still face a critical trade-off: methods with strong contextual or global reasoning capabilities (e.g., Transformer-based models) often suffer from high computational overhead and poor edge deployability, while efficient CNN-based detectors (e.g., YOLO variants) typically lack explicit multi-scale context awareness and robust spatial attention for small, camouflaged targets in UAV imagery. To bridge this gap, YOLO-WL integrates MSDDSC for hierarchical context enrichment and MLKSA, a lightweight yet powerful multi-scale spatial attention module that jointly expands receptive fields, validates cross-scale consistency, and suppresses scale-varying background clutter—without sacrificing inference speed.

To better illustrate the distinctions between our approach and representative prior works, we summarize their key characteristics in Table 1.

## 3. Methodology

### 3.1. YOLO-WL Model

YOLOv8 consists of four key modules: an input interface, a backbone for feature extraction, a neck for multi-scale feature fusion, and a detection head for generating final predictions. Owing to a series of well-designed optimizations, it achieves notable gains in both performance and adaptability. To meet diverse application requirements and computational limitations, YOLOv8 offers five scaled variants—n, s, m, l, and x—enabling flexible deployment. This work addresses UAV-based wildlife recognition by building upon the lightweight YOLOv8n model, which serves as the baseline for our proposed enhancements.

**Figure 1:**
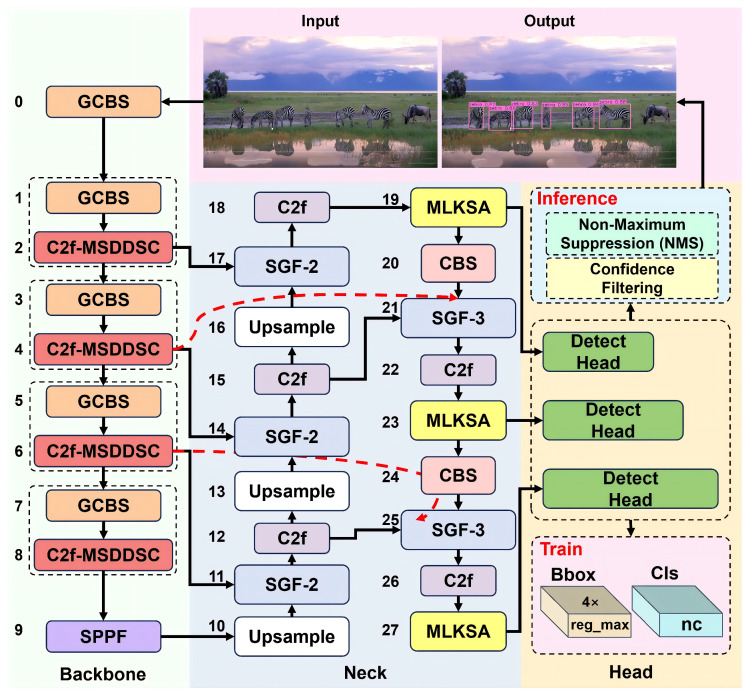
Network architecture of YOLO-WL. GCBS denotes group convolution, batch normalization, and SiLU activation function.

This paper introduces an MSDDSC module, seamlessly integrated with the C2f structure to replace the original C2f blocks in the YOLOv8n backbone. A novel SSA-PAN network is designed and incorporated into the feature fusion stage. Furthermore, an MLKSA mechanism is added between the neck and the detection head. We also propose a Layer-wise Feature Compression (LFC) strategy to reduce the channel dimension at stride = 32 from 512 to 256, specifically applied to layers 7–9. Based on these enhancements, we develop an efficient UAV-based wildlife recognition model named YOLO-WL. The overall architecture and module designs are illustrated in Figure 1, with detailed network configurations provided in Table 2.

### 3.2. MSDDSC Module

Wildlife recognition from drone imagery is challenged by low discriminability of species-specific features. Closely related species or individuals at different life stages often appear highly similar, making visual differentiation difficult. Meanwhile, aerial perspectives limit the observation of subtle behavioral cues due to distance and viewing angles, further compromising identification accuracy and reliability.

To address this issue, we propose the MSDDSC module. The module integrates rich contextual information, effectively combining local details with global structural features. This enhances the model’s ability to distinguish between visually similar species. Specifically, the multi-scale design enables the extraction of key features across different spatial ranges, improving the differentiation of morphologically similar individuals. At the same time, the dilated depthwise separable convolution expands the receptive field without increasing computational cost, enabling the extraction of richer semantic information. The integration of local and global information not only improves the model’s sensitivity to subtle morphological differences but also enhances its ability to interpret and utilize animal behavioral patterns from aerial viewpoints.

**Figure 2:**
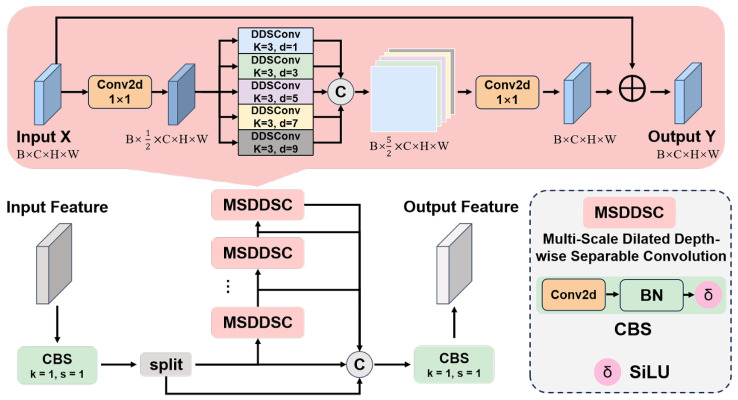
The structure of C2f-MSDDSC module.

We then integrate MSDDSC into the lightweight C2f block of YOLOv8, forming the C2f-MSDDSC module (Figure 2). This design preserves the original efficiency of C2f while significantly improving discrimination of visually similar wildlife species, reducing both parameters and FLOPs, and maintaining detection performance.

Let the input feature map be denoted as $X\in R^{B\times C\times H\times W}$, where *B* is the batch size, *C* represents the number of input channels, and (H,W) denotes the spatial dimensions. The MSDDSC module performs multi-scale feature fusion through the following steps:

First, a 1×1 convolutional layer is applied to compress the input feature map *X* along the channel dimension:

$$X^{\prime }=\sigma \left(BN\left(Conv_{1\times 1}\left(X\right)\right)\right)\in R^{B\times C^{\prime }\times H\times W}$$

Here, $C^{\prime }$ denotes the number of channels after compression, where $C^{\prime }=\left\lfloor e\cdot C\right\rfloor$ and *e* is the compression ratio.

Secondly, a set of pre-defined dilation rates $R=\{r_{1},r_{2},\ldots ,r_{n}\}$ is employed. For each dilation rate $r_{i}\in R$, a depthwise separable convolution is performed in parallel:

$$DDWConv_{k\times k}^{d=r_{i}}{\left(X^{\prime }\right)}_{c,i,j}=\sum_{m=0}^{k-1}\sum_{n=0}^{k-1}W_{\left(m,n\right)}^{\left(c\right)}\cdot X_{c,i+r_{i}\cdot m,j+r_{i}\cdot n}^{\prime }$$

$$Z^{\left(i\right)}=\sigma \left(BN\left(Conv_{1\times 1}\left(DDWConv_{k\times k}^{d=r_{i}}\left(X^{\prime }\right)\right)\right)\right),\;\forall r_{i}\in R$$

In the equation, $W^{\left(c\right)}\in R^{k\times k}$ denotes the convolutional kernel weight corresponding to channel *c*, and *k* represents the kernel size (in this case, k=3).

Then, all output feature maps from the parallel branches are concatenated along the channel dimension:

$$Z=Concat\left(Z^{\left(1\right)},Z^{\left(2\right)},\ldots ,Z^{\left(k\right)}\right)\in R^{B\times kC^{\prime }\times H\times W}$$

An additional 1×1 convolutional layer is used to integrate the multi-scale features:

$$\hat{Y}=\sigma \left(BN\left(Conv_{1\times 1}\left(Z\right)\right)\right)\in R^{B\times C^{\prime }\times H\times W}$$

Finally, the output feature map $Y\in R^{B\times C\times H\times W}$ is obtained by performing an element-wise addition between the integrated feature $\hat{Y}$ and the original input feature map *X*:

$$Y=\hat{Y}+X$$

### 3.3. MLKSA Mechanism

In UAV-based wildlife recognition, multi-source environmental interferences significantly challenge system robustness and accuracy. Complex terrain and occlusion cause incomplete targets or feature loss, while dynamic lighting degrades image contrast and feature consistency. High similarity between animals and background elements (e.g., rocks, leaves, trunks) further increases false positives. These factors interact synergistically, complicating algorithm design and optimization.

**Figure 3:**
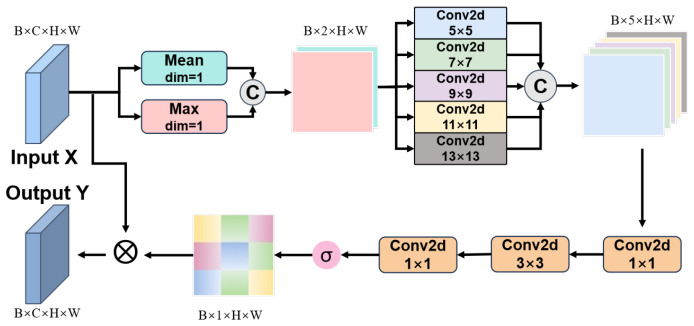
Illustration of MLKSA module. σ(·) indicates the sigmoid activation function.

To address these challenges, we propose the MLKSA mechanism (Figure 3), which integrates multi-scale feature extraction with large-kernel convolutions. This enables dynamic focus on key regions across spatial scales, effectively suppressing environmental interference. Specifically, the kernel sizes of 5/7/9/11/13 are carefully chosen to match the typical scale distribution of wildlife targets in UAV imagery, ensuring continuous and complementary coverage from fine details to global context. This multi-scale design not only captures targets of varying sizes but also suppresses background clutter at their native resolutions through cross-scale consistency validation. MLKSA enhances detailed texture capture in close-ups and holistic perception of distant small targets, expanding the receptive field for better foreground-background distinction. A spatial attention mechanism further improves semantic region representation while suppressing irrelevant background information, significantly boosting recognition accuracy and robustness in UAV-based wildlife scenarios.

Let $X\in R^{B\times C_{1}\times H\times W}$ be the input feature map with batch size *B*, channels $C_{1}$, and spatial dimensions H×W. The model computes the output $Y\in R^{B\times C_{2}\times H\times W}$.

First, two types of global statistical features are extracted along the channel dimension of the input *X*:

$$X_{\mathrm{mean}}=\frac{1}{C}\sum_{c=1}^{C}X_{:,c,:,:},\;X_{\max}=\max_{c}X_{:,c,:,:}$$

These two types of features are then concatenated along the channel dimension to form the initial fused feature:

$$Z=\left[X_{\mathrm{mean}},X_{\max}\right]\in R^{B\times 2\times H\times W}$$

Secondly, a set of pre-configured large-sized convolutional kernels is defined as $\kappa =\{k_{1},k_{2},k_{3},k_{4},k_{5}\}$, where $k_{i}$ is typically set to a relatively large value (in this work, we set κ={5,7,9,11,13}). The concatenated feature *Z* is processed by parallel convolutional branches, each with a distinct kernel size, to capture spatial features across multiple scales.

$$F_{k_{i}}=ReLU\left(BN\left(Conv_{k_{i}\times k_{i}}\left(Z\right)\right)\right),\;\forall k_{i}\in \kappa$$

Here, $F_{k_{i}}\in R^{B\times 1\times H\times W}$ denotes the feature map extracted using the kernel $k_{i}\times k_{i}$.

Subsequently, the multi-scale feature maps $\left\{F_{k_{1}},F_{k_{2}},F_{k_{3}},F_{k_{4}},F_{k_{5}}\right\}$ from the parallel branches are fused channel-wise to yield a consolidated feature representation:

$$F_{\mathrm{cat}}=Concat\left(F_{k_{1}},F_{k_{2}},F_{k_{3}},F_{k_{4}},F_{k_{5}}\right)\in R^{B\times 5\times H\times W}$$

To effectively fuse and refine the multi-scale information, two consecutive convolutional layers are employed:

$$F^{\prime }=ReLU\left(BN\left(Conv_{1\times 1}\left(F_{\mathrm{cat}}\right)\right)\right)$$

$$F^{\prime \prime }=ReLU\left(BN\left(Conv_{3\times 3}\left(F^{\prime }\right)\right)\right)$$

Here, $F^{\prime }\in R^{B\times 5\times H\times W}$, and $F^{\prime \prime }\in R^{B\times 1\times H\times W}$.

Finally, a standard 1×1 convolutional layer is applied to map the fused feature $F^{\prime \prime }$ into a spatial attention weight map *A*, which is then element-wise multiplied with the original input feature map *X* to produce the final output feature map *Y*:

$$A=\sigma \left(Conv_{1\times 1}\left(F^{\prime \prime }\right)\right)\in {\left[0,1\right]}^{B\times 1\times H\times W}$$

Y=X⊙A

### 3.4. SSA-PAN Network

In drone-captured aerial scenes, wildlife targets typically occupy only a few pixels due to the high altitude of image acquisition. This results in extremely small object sizes and blurred visual details, making it difficult to effectively extract and recognize critical visual features. At the same time, existing detection methods often rely heavily on high-level semantic features (e.g., P3–P5) for multi-scale feature fusion, while neglecting the rich spatial detail information present in shallow layers. Such fusion mechanisms lack explicit spatial guidance, leading to insufficient alignment and complementarity between features from different levels, which in turn results in suboptimal cross-scale feature integration. Particularly under complex background conditions and low-resolution inputs, the spatial perception capability of the model becomes limited, significantly degrading its performance in locating and recognizing tiny objects.

**Figure 4:**
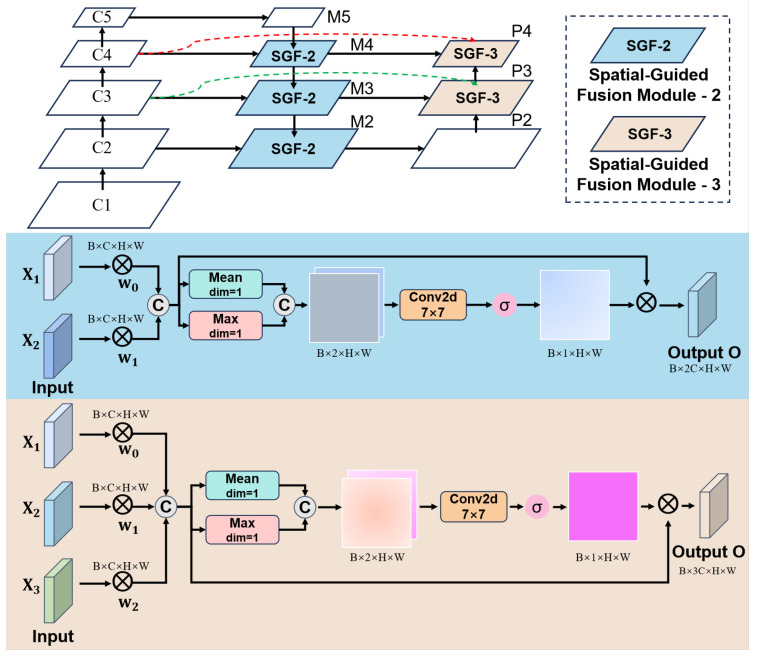
SSA-PAN network schematic.

To address the challenges of tiny object size, insufficient feature representation, and inadequate multi-scale feature fusion in wildlife detection from drone-captured imagery, this paper proposes the SSA-PAN network, as illustrated in Figure 4. The proposed method introduces a SGF module based on the shallow feature pyramid (P2–P4). By performing more precise spatial alignment and information fusion of multi-scale features at an early stage, the model enhances its spatial perception capability for tiny targets. Compared to traditional FPN approaches that primarily rely on high-level semantic features (e.g., P3–P5) for feature fusion, the proposed SSA-PAN places greater emphasis on leveraging the rich spatial details present in shallow layers. Furthermore, the spatial-guided information flow improves the complementarity between features across different levels, thereby effectively alleviating the difficulties in recognition caused by small object size and limited image resolution.

## 4. Experimental Setup

### 4.1. Dataset

To comprehensively evaluate the effectiveness and generalization capability of the proposed method in complex scenarios, we conducted systematic experiments and comparative analyses on multiple publicly available datasets. The main experiments were carried out using the WAID (Wildlife Aerial Images from Drone) dataset [50], released by Beijing Forestry University’s College of Information Science. This dataset was extensively utilized in our study to validate the practical performance of the proposed algorithm in real-world applications.

**Figure 5:**
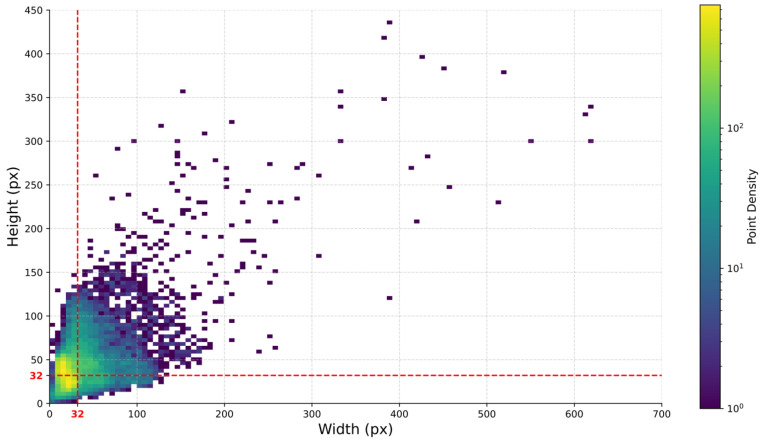
Distribution of target object sizes within the WAID dataset.

The WAID dataset is currently among the largest, multi-category, and high-quality aerial image datasets specifically designed for wildlife monitoring using UAV platforms. It comprises six representative wildlife categories, namely sheep, cattle, seals, camels, kiangs, and zebras, and covers a variety of typical habitat environments, including deserts, grasslands, and beaches. The dataset contains a total of 14,366 UAV-captured images collected from diverse geographical regions and environmental conditions, which exhibit strong scene complexity and realistic detection challenges. These images are partitioned into three subsets: 10,056 for training, 2873 for validation, and 1437 for testing, with detailed class statistics presented in Table 3. The size distribution of object instances in the training set is shown in Figure 5. Statistical results indicate that the majority of target objects have pixel dimensions smaller than 32 × 32, suggesting a high proportion of small-scale targets. This characteristic aligns well with the focus and objectives of this study.

Additional experiments on the Aerial Sheep and AI-TOD datasets [51] validate the generalization capability of the proposed approach.

The Aerial Sheep dataset was constructed by Riis in June 2022 and publicly released on the Roboflow Universe platform. It is intended to advance research and applications of object detection algorithms for sheep in UAV-captured imagery. The dataset comprises a total of 4133 preprocessed and data-augmented aerial images, consisting of 3609 training images, 350 validation images, and 174 test images. Detailed statistical information is provided in Table 4. Due to variations in weather and lighting conditions, image quality exhibits significant fluctuations, which substantially increases the difficulty of object detection. Additionally, the sheep instances vary widely in scale and pose, further complicating target recognition and localization.

The AI-TOD dataset is a large-scale benchmark specifically designed for tiny object detection in aerial imagery. It contains 28,036 images, divided into 11,214 training, 2804 validation, and 14,018 test images. These images cover over 700,000 annotated object instances across eight common aerial object categories, such as airplanes, vehicles, and pedestrians, as detailed in Table 5. The average object size in AI-TOD is only 12.8 pixels, which is significantly smaller than that in most other aerial datasets, making it particularly challenging. This dataset provides a rigorous evaluation of the proposed method’s robustness and its ability to detect small objects.

### 4.2. Implementation Details and Evaluation Metrics

To comprehensively evaluate the performance of the proposed algorithm, a standardized experimental environment was established. All experiments were conducted on the CentOS 8.5.2 operating system, with the neural network models implemented using the PyTorch deep learning framework. The YOLOv8n model was selected as the baseline for comparative analysis. Detailed hardware and software configurations are presented in Table 6.

To ensure fair comparison of experimental results, all improved models were trained using an identical set of hyperparameters, and no pre-trained weights were utilized throughout the training process. The detailed hyperparameter configurations are summarized in Table 7.

To comprehensively evaluate the proposed model, we adopt standard metrics: Precision (P), Recall (R), mean Average Precision (mAP), parameter count, and GFLOPs. Precision reflects the reliability of positive detections, whereas Recall indicates the fraction of ground-truth instances successfully retrieved. mAP—computed as the average of per-class AP values, each derived from the area under its precision-recall curve—serves as the primary indicator of overall detection accuracy. Meanwhile, parameter count and GFLOPs characterize model compactness and computational load, respectively, bo th of which are pivotal for deployment on edge devices with limited resources.

$$\mathrm{Precision}=\frac{TP}{TP+FP}$$

$$\mathrm{Recall}=\frac{TP}{TP+FN}$$

$$AP=\int_{0}^{1}P\left(R\right)dR$$

$$mAP=\frac{1}{K}\sum_{k\in K}AP\left(k\right)$$

In this context, TP, FP, and FN denote true positives (correctly detected ground-truth objects), false positives (background regions wrongly detected as objects), and false negatives (ground-truth objects that are missed), respectively.

## 5. Comparative Experiments

### 5.1. Comparison Experiments of Different Attention Mechanisms

To evaluate the MLKSA attention mechanism, state-of-the-art attention modules were integrated into YOLOv8n and compared on the WAID test set. Results are summarized in Table 8.

As shown in Table 8, several existing attention modules (e.g., SEv1, ECA, CBAM) fail to improve YOLOv8n’s performance and even reduce mAP slightly, while others offer only marginal gains. In contrast, the proposed MLKSA module achieves significant improvements with minimal increases in parameters and computational cost. It reaches an mAP@0.5 of 93.2% (+0.6%) and mAP@0.5:0.95 of 57.4% (+0.5%), demonstrating strong effectiveness and robustness in drone-based small-object detection, with high potential for real-world applications.

### 5.2. Comparison Experiment of Shallow Feature Fusion Methods

**Figure 6:**
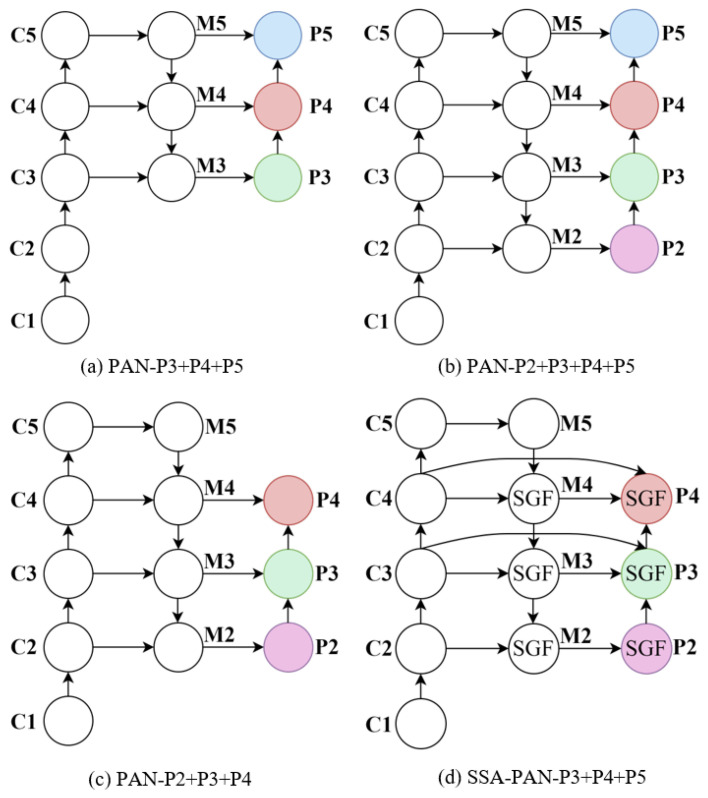
Comparative results of various feature fusion strategies.

To evaluate the effectiveness of SSA-PAN in multi-scale feature fusion, comparative experiments were conducted with different fusion strategies on the WAID test set (results in Table 9). Two factors were analyzed: (1) replacing the high-level P5 layer with the shallow P2 layer for improved small-object detection (Figure 6a–c), and (2) comparing the proposed SSA-PAN architecture against the conventional PAN structure (Figure 6d).

As shown in Table 9, the original YOLOv8n model employs a PAN structure comprising feature levels P3, P4, and P5 for multi-scale feature fusion, achieving an mAP@0.5 of 92.6% and an mAP@0.5:0.95 of 56.9% on the test set. When the high-level semantic feature layer P5 was replaced with the higher-resolution shallow feature layer P2, forming a new PAN structure based on P2+P3+P4, the model demonstrated significant improvements in small-object detection performance. Specifically, the mAP@0.5 and mAP@0.5:0.95 increased to 95.0% and 59.7%, respectively, while Precision and Recall also improved to 93.2% and 90.2%. These results indicate that incorporating high-resolution shallow features enhances the model’s ability to capture fine-grained details of small objects, thereby significantly improving overall detection performance.

However, incorporating features from levels P2, P3, P4, and P5 using the P2+P3+P4+P5 PAN structure resulted in a slight decline in detection performance compared to the P2+P3+P4 configuration. This observation suggests that the inclusion of high-level semantic feature layers—originally designed for large-object detection—along with excessive feature fusion stages, may introduce redundant information that interferes with the model’s ability to learn discriminative features for small objects, thereby degrading overall detection accuracy. In contrast, the proposed SSA-PAN feature fusion network, based on the P2+P3+P4 architecture, demonstrates a more rational structural design and enhanced practical effectiveness in multi-scale feature integration. With only a marginal increase in computational cost, the mAP@0.5 and mAP@0.5:0.95 are improved to 95.2% and 59.8%, respectively, further validating its effectiveness and superiority in small-object detection tasks within the context of wildlife recognition from drone-based perspectives.

### 5.3. Ablation Experiments

To evaluate the contribution of each proposed module in drone-based wildlife detection, an ablation study was conducted by incrementally integrating components into the YOLOv8n baseline. The modules include C2f-MSDDSC, MLKSA attention, SSA-PAN, and lightweight elements LFC and GConv. Results are summarized in Table 10.

As shown in Table 10, in the single-module ablation experiments, the original baseline model achieves an mAP@0.5 of 92.6% and an mAP@0.5:0.95 of 56.9% on the test set. When only the C2f-MSDDSC module is introduced to construct Model1, the model parameters increase by 0.4M, while the computational cost slightly decreases by 0.7 GFLOPs. The mAP@0.5 and mAP@0.5:0.95 improve to 93.2% and 56.9%, respectively. This indicates that the module enhances the model’s ability to distinguish morphologically similar categories by effectively modeling contextual information and enabling the synergistic fusion of local fine-grained features with global structural information. In Model2, which incorporates only the MLKSA module, the detection performance improves significantly with almost no increase in model parameters or computational cost. Specifically, the mAP@0.5 reaches 93.2%, and the mAP@0.5:0.95 increases to 57.4%. This demonstrates that the proposed multi-scale large-kernel spatial attention mechanism effectively enhances the model’s spatial perception capability and exhibits strong robustness in suppressing complex background noise. In contrast, Model3, which includes only the SSA-PAN module, achieves superior performance despite a 1.0 M reduction in parameters and an increase of 3.6 GFLOPs in computational cost. It attains an mAP@0.5 of 95.2% and an mAP@0.5:0.95 of 59.8%, significantly outperforming the other two individual improvements. This result further confirms that SSA-PAN emphasizes the rich spatial details in shallow features and improves cross-level feature complementarity through effective spatial guidance, thereby substantially enhancing the overall detection performance.

In the multi-module ablation experiments, Model4, which integrates both the C2f-MSDDSC and MLKSA modules, achieves an mAP@0.5 of 93.9% and an mAP@0.5:0.95 of 58.4%, indicating that the combination enhances both contextual semantic modeling and spatial perception while effectively suppressing background noise. Model6, which combines C2f-MSDDSC with SSA-PAN, achieves the best performance among all module combinations, with an mAP@0.5 of 96.1% and an mAP@0.5:0.95 of 60.9%. This result further illustrates the significant synergistic and complementary advantages of semantic detail fusion and shallow feature enhancement strategies in small-object detection tasks.

Finally, based on the integration of multiple modules, lightweight design is introduced through the LFC operation and GConv to compress and optimize the model. Compared to the baseline, the proposed YOLO-WL reduces the total number of parameters by 53.3% and shrinks the model size to 45.9% of the original. Although the computational cost increases by 18.3%, the detection performance remains strong, with mAP@0.5 and mAP@0.5:0.95 reaching 94.2% and 58.0%, respectively—representing improvements of 1.6% and 1.1% over the baseline model. These results clearly demonstrate that YOLO-WL not only enhances detection accuracy but also significantly improves model compactness and deployment feasibility, making it highly suitable for real-world applications.

**Figure 7:**
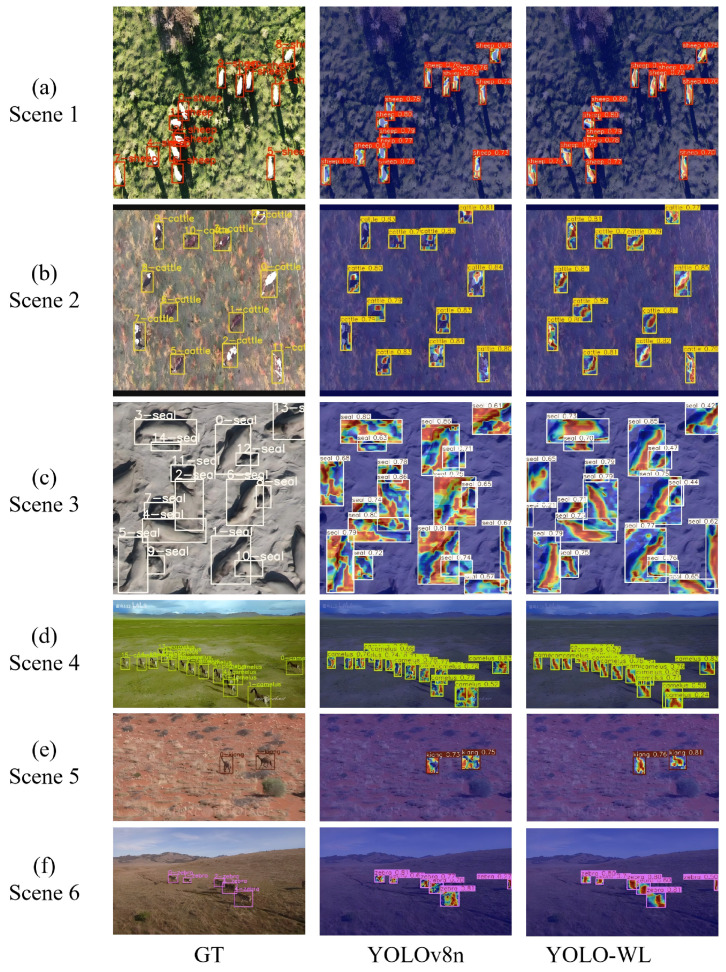
Visual comparison results of YOLOv8n and YOLO-WL across multiple scenes.

To intuitively compare feature perception before and after improvement, Grad-CAM visualizations of YOLOv8n and YOLO-WL are presented in Figure 7 for six representative scenarios. Redder colors indicate higher attention, reflecting the model’s focus on target localization.

As shown in the visualization results in Figure 7, the proposed YOLO-WL demonstrates a stronger capability than the baseline model YOLOv8n in accurately capturing the morphological structures of wildlife. It achieves enhanced perception and modeling of target contours, and maintains robust performance even under complex background interference.

To provide a more comprehensive evaluation of the performance improvements achieved through the proposed model enhancements, this study also summarizes the detection results across different object categories before and after the modifications, as shown in Table 11.

As shown in Table 11, compared to the baseline model YOLOv8n, the proposed YOLO-WL achieves the most notable performance improvements on the Camelus, Kiang, and Zebra categories. Specifically, the mAP@0.5 increases by 2.7%, 3.2%, and 2.2%, respectively, while the mAP@0.5:0.95 improves by 2.3%, 1.6%, and 2.1%. In contrast, a slight performance degradation is observed on the Seal category, with mAP@0.5 and mAP@0.5:0.95 decreasing by 0.3% and 0.5%, respectively. Nevertheless, the overall performance across all four evaluation metrics shows consistent gains, which further confirms the effectiveness of the proposed model enhancements.

Moreover, when deployed on the RK3588 NPU, the model achieves a real-time inference speed of 72.1 FPS, demonstrating computational efficiency sufficient to support high-frame-rate real-time detection systems (which typically require an end-to-end frame rate of at least 30 FPS). This shows that YOLO-WL significantly improves detection accuracy without sacrificing real-time performance, effectively balancing precision and efficiency, and thus exhibits strong potential for edge deployment and practical applications.

### 5.4. The Impact of Training Dataset Scale on YOLO-WL Performance

To systematically investigate the performance of the proposed YOLO-WL algorithm under varying training set sizes, this paper randomly samples subsets of 20%, 40%, 60%, and 80% from the full training set and conducts separate training and evaluation on each. The experimental results are shown in Table 12.

As shown in Table 12, as the training set ratio increases from 20% to 100%, all performance metrics of YOLO-WL steadily improve, indicating that the model exhibits strong scalability with respect to data volume. Notably, when the training ratio increases from 20% to 40%, mAP@0.5 improves significantly by 12.9 percentage points, demonstrating that adding data in the early stages yields the most pronounced performance gains. Even with only 20% of the training data, YOLO-WL achieves an mAP@0.5 of 75.6%, showcasing its robust few-shot learning capability.

**Figure 8:**
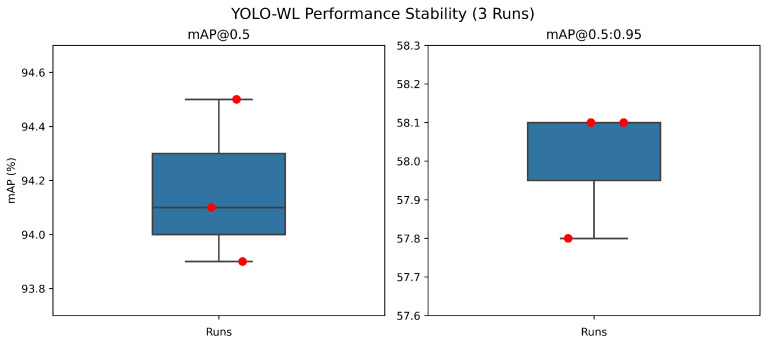
Stability of YOLO-WL across repeated runs.

Moreover, all experimental results are reported as the average of three independent runs. Specifically, when trained on the full dataset, the variances of the model’s accuracy are 0.0622 for mAP@0.5 and 0.0200 for mAP@0.5:0.95. This demonstrates that YOLO-WL exhibits high stability across multiple training and evaluation runs, with minimal performance fluctuation. Corresponding boxplots are provided in Figure 8.

### 5.5. Model Prediction Performance Across Multiple Scenarios

#### 5.5.1. Robust Detection Under Favorable Conditions

**Figure 9:**
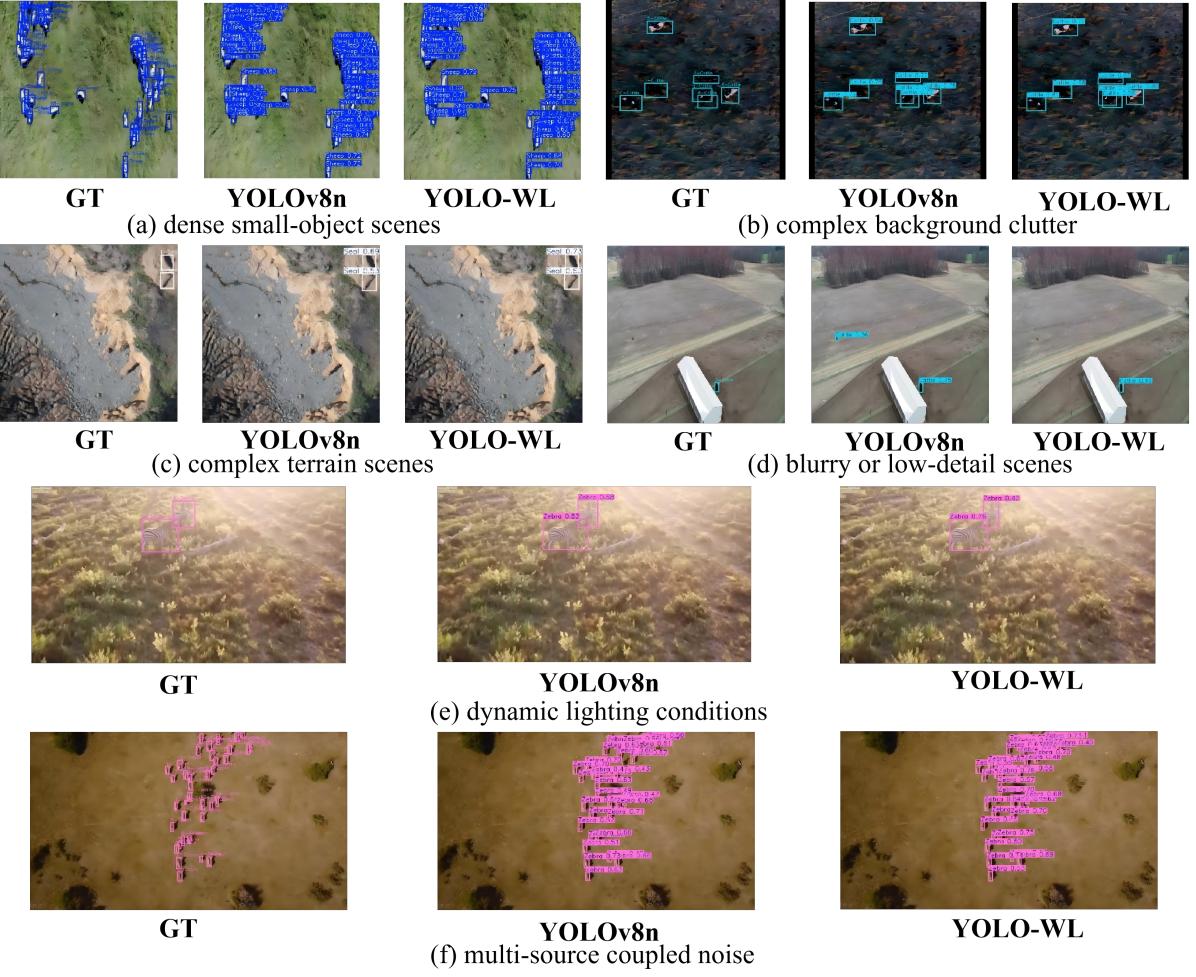
Robust detection with YOLO-WL under challenging conditions.

To comprehensively evaluate the model’s generalization capability and robustness in complex real-world scenarios, we conduct a comparative evaluation between YOLO-WL and the baseline model YOLOv8n under six representative challenging conditions: (a) dense small-object scenes, (b) complex background clutter, (c) complex terrain environments, (d) blurry or low-detail scenes, (e) dynamic lighting conditions, and (f) multi-source coupled noise interference. The qualitative results are shown in Figure 9.

As shown in Figure 9, the proposed YOLO-WL network accurately and consistently detects wildlife targets across a variety of complex scenarios. Even under highly challenging imaging conditions, the method effectively suppresses false positives and missed detections, maintaining high localization accuracy and classification confidence, which clearly demonstrates its excellent robustness and adaptability to diverse environmental conditions.

#### 5.5.2. Failure Modes and Challenging Scenarios

**Figure 10:**
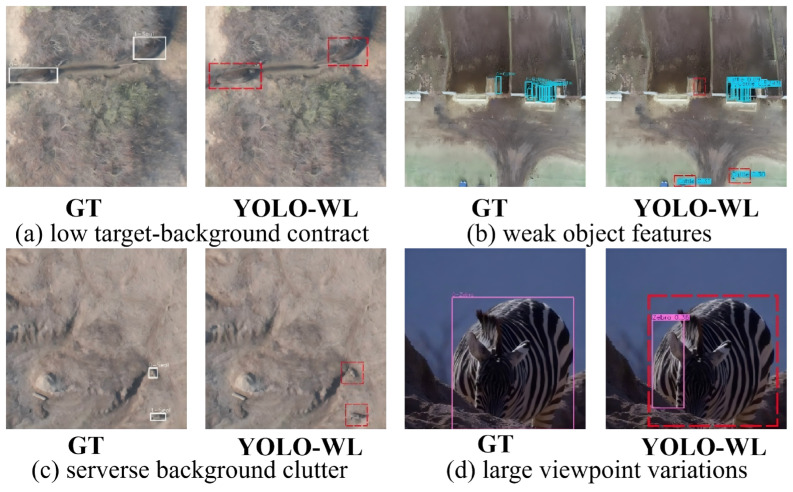
Failure cases and limitations of YOLO-WL.

Although YOLO-WL demonstrates strong performance across most complex scenarios, several challenging failure cases remain. As shown in Figure 10 (highlighted with red boxes), the model may exhibit noticeable false positives or missed detections under the following conditions: (1) drastic viewpoint changes causing severe object deformation; (2) high visual similarity between the ground background and target animals in terms of color and texture, leading to significant ambiguity; and (3) extremely weak target features—such as ultra-low resolution, occlusion due to posture, or motion blur—that provide insufficient discriminative cues. Despite these limitations, YOLO-WL still significantly outperforms existing methods overall, with its failures primarily occurring in extreme edge cases that reflect common technical challenges in UAV-based wildlife detection.

### 5.6. Comparative Experiment of Advanced Algorithm on the WAID Dataset

To comprehensively evaluate YOLO-WL’s performance in drone-based wildlife detection, we compare it with mainstream lightweight models including YOLOv3-tiny [48], YOLOv5n [49], YOLOv6n [50], YOLOv7-tiny [51], and YOLOv8n [52] to YOLOv12n [53]. All models are trained and evaluated on the WAID dataset under identical settings—consistent training strategy, data pipeline, and evaluation metrics—to ensure fair and objective comparison. Results are summarized in Table 13.

**Figure 11:**
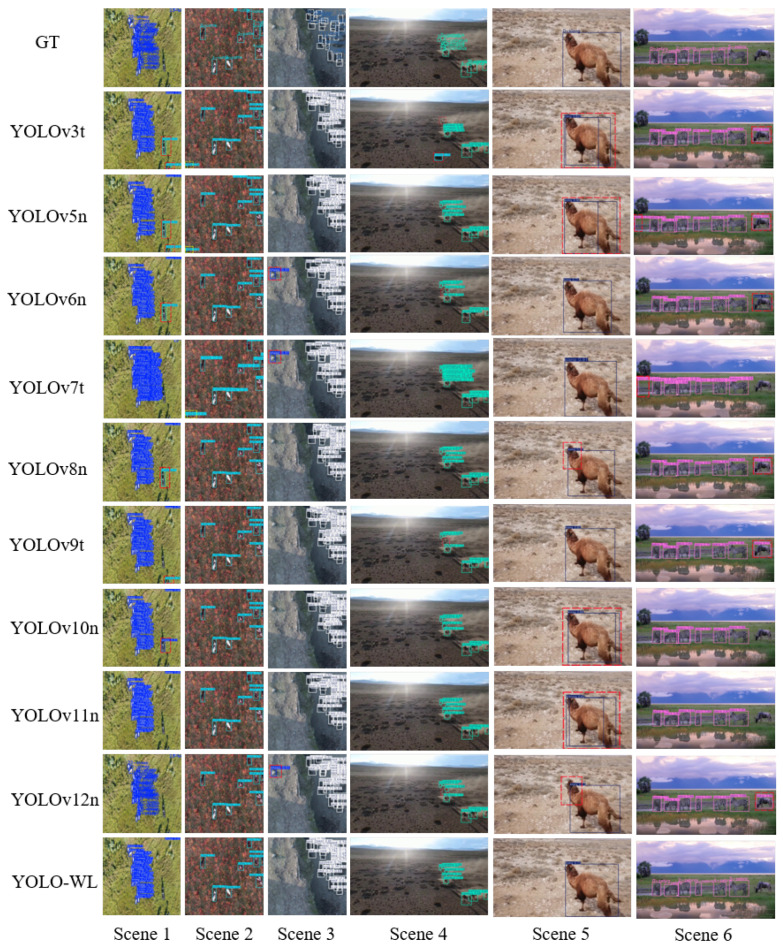
Visualization of comparison results across different scenarios in the WAID dataset.

As shown in Table 13, YOLO-WL achieves the highest mAP@0.5 and mAP@0.5:0.95 scores of 94.2% and 58.0%, respectively, outperforming all compared models and demonstrating superior detection accuracy. Compared to the baseline YOLOv8n, YOLO-WL reduces model parameters and model size by 53.3% and 45.9%, respectively, with only an 18.3% increase in computational cost, while still achieving improvements of 1.7% and 1.2% in mAP@0.5 and mAP@0.5:0.95. These results indicate that YOLO-WL not only significantly reduces model complexity and deployment cost but also enhances detection performance, effectively balancing accuracy and efficiency for practical deployment. To offer a more intuitive visualization of the detection performance of various algorithms on the WAID dataset, six representative scenarios are selected for a visual comparison of detection results across different models. The detailed prediction outcomes are shown in Figure 11, where dashed bounding boxes highlight regions with major false positives and false negatives observed during detection.

As illustrated in Figure 11, objects in the WAID dataset not only exhibit considerable scale variations but also show significant appearance differences under drone-captured viewpoints. Moreover, complex backgrounds containing distractors further complicate the task of target recognition. Under such challenging conditions, the YOLO-WL algorithm exhibits more comprehensive and accurate detection performance across all object categories. It effectively captures target features under multi-scale and multi-viewpoint settings, thereby demonstrating enhanced environmental adaptability and robustness in object detection.

## 6. Generalization Experiments

### 6.1. Performance Comparison on the Aerial Sheep Dataset

To validate the applicability and generalization capability of the YOLO-WL algorithm in drone-based wildlife detection tasks, comparative experiments are conducted against several mainstream object detection algorithms using the Aerial Sheep dataset. The results on the test set are summarized in Table 14.

The experimental results in Table 14. demonstrate that YOLO-WL achieves excellent detection performance on the Aerial Sheep dataset, achieving mAP@0.5 and mAP@0.5:0.95 scores of 98.1% and 61.7%, respectively. These results significantly outperform those of other lightweight object detection models, indicating strong generalization capability. Compared to the baseline model YOLOv8n, YOLO-WL improves by 0.6 and 0.2 percentage points on these two metrics, highlighting its superior detection performance in scenarios involving densely distributed small targets.

**Figure 12:**
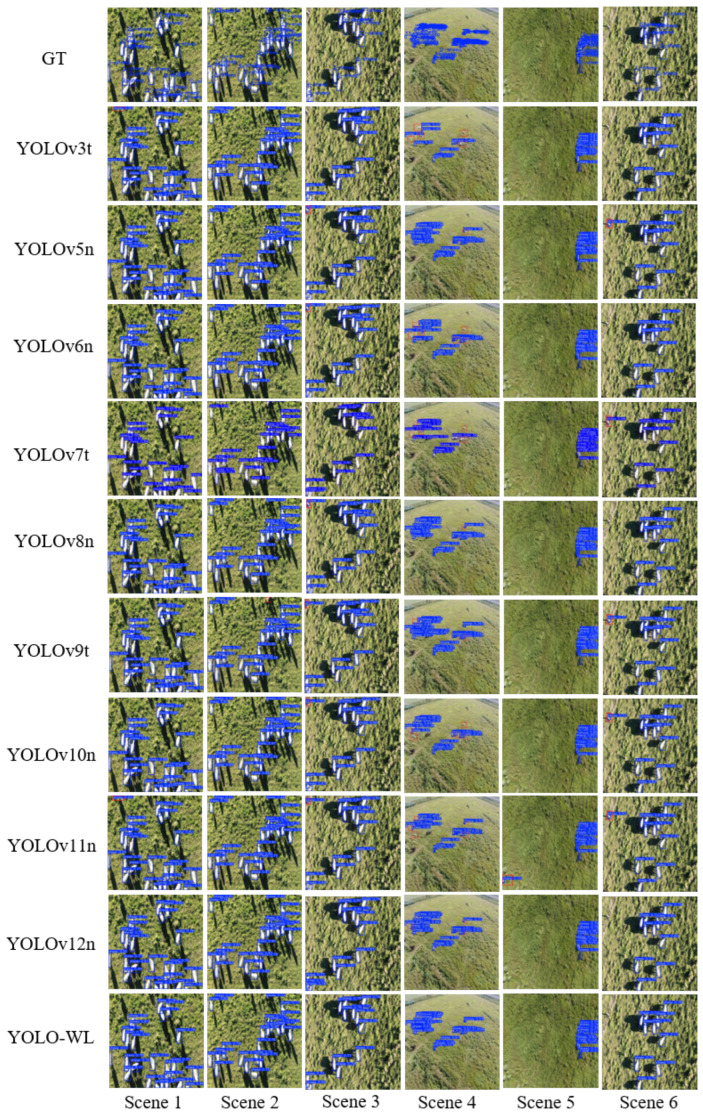
Visualization of comparison results across different scenarios in the Aerial Sheep dataset.

To offer a more intuitive comparison of the detection performance of various algorithms on the Aerial Sheep dataset, six typical scenarios are selected to visualize the detection results of each model. The detailed detection results are shown in Figure 12.

As shown in Figure 12, in the Aerial Sheep dataset, sheep are densely distributed and exhibit small sizes, while the grassland background contains many distractors similar in appearance to the targets, posing significant challenges for accurate detection. In contrast, the YOLO-WL algorithm achieves superior performance by accurately localizing all targets, demonstrating enhanced robustness. In comparison, other algorithms suffer from various levels of false negatives and false positives in this scenario, indicating relatively limited detection capabilities.

### 6.2. Performance Comparison on the AI-TOD Dataset

Similarly, on the AI-TOD dataset, comparative experiments are conducted with several mainstream object detection algorithms. The experimental results, as shown in Table 15, further validate the proposed method’s generalization capability and detection performance in complex aerial scenarios.

The experimental results in Table 15 show that YOLO-WL continues to outperform existing lightweight object detection models on the AI-TOD dataset. It achieves mAP@0.5 and mAP@0.5:0.95 scores of 32.3% and 14.0%, respectively, which represent the best performance among the compared lightweight models. Compared to the baseline YOLOv8n, YOLO-WL improves these metrics by 1.9 and 1.0 percentage points, further demonstrating its superior detection performance in complex aerial scenarios. These results indicate that YOLO-WL achieves higher accuracy and better generalization when handling drone-captured images with dense small objects and significant scale variations.

**Figure 13:**
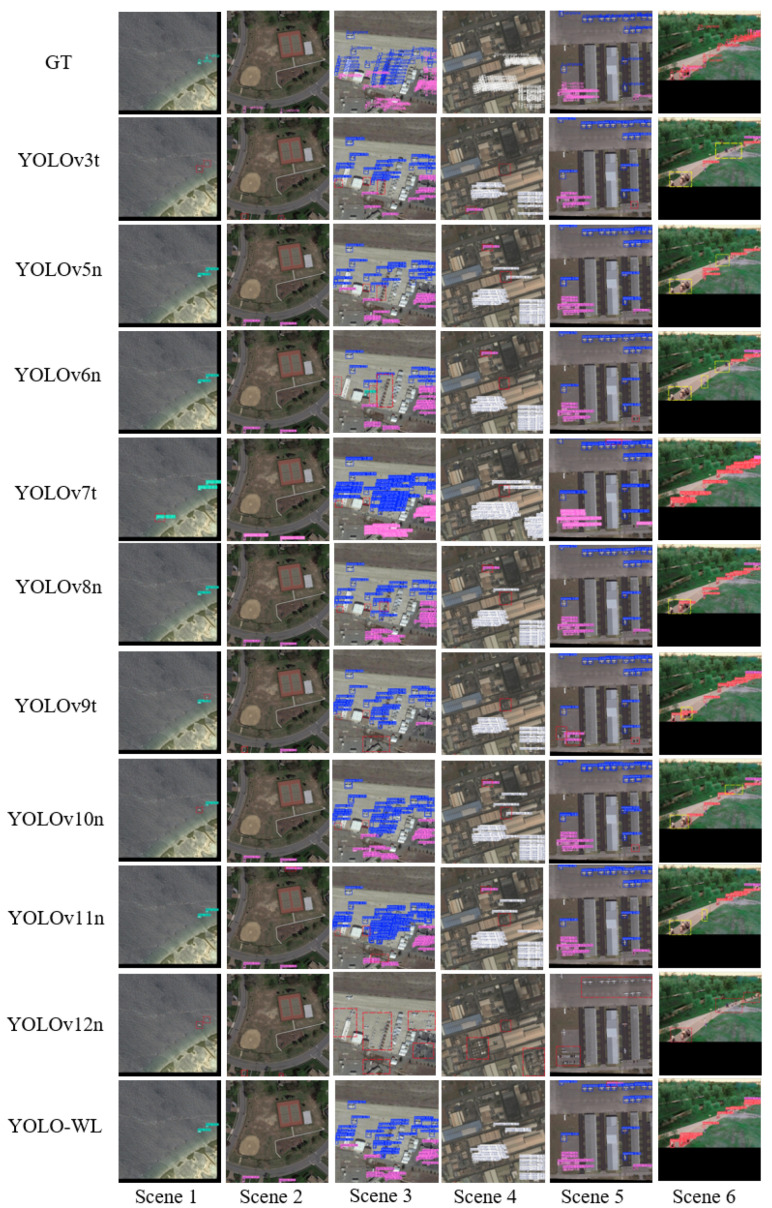
Visualization of comparison results across different scenarios in the AI-TOD dataset.

To provide a more comprehensive evaluation of the proposed method’s adaptability in complex aerial scenarios, we select six representative scenes from the AI-TOD dataset to visually compare the detection performance of different models. The detailed results are presented in Figure 13.

As can be observed from Figure 13, objects of different categories in the AI-TOD dataset are situated in environments with significant variations, resulting in a variety of complex background interferences. Additionally, due to the small size of the targets, their features are difficult to extract and learn effectively. Under such challenging conditions for small-object detection, other mainstream object detection algorithms exhibit varying degrees of missed detections or false positives across different environments. In contrast, the YOLO-WL algorithm demonstrates more comprehensive and accurate detection capabilities across all object categories, reflecting its superior robustness and adaptability.

## 7. Conclusions

This paper presents YOLO-WL, a wildlife detection algorithm designed for UAV-captured aerial imagery, to address the challenges posed by visually similar species, complex environmental interference, and the small size of target animals. To enhance feature representation, we design the MSDDSC module and construct the C2f-MSDDSC structure, enabling the integration of fine-grained local details with broader contextual information. To improve robustness against multi-source background disturbances, we introduce the MLKSA mechanism, which adaptively emphasizes salient animal regions across multiple scales. Furthermore, we propose the SSA-PAN framework, which achieves precise spatial alignment and complementary fusion of shallow features, thereby improving detection accuracy for tiny wildlife targets. Experimental evaluations on the WAID, Aerial Sheep, and AI-TOD datasets demonstrate that YOLO-WL surpasses state-of-the-art lightweight object detection models in both accuracy and robustness, while also exhibiting strong generalization ability across diverse scenarios.

However, despite its strong cross-scenario generalization on known categories, YOLO-WL’s training relies on large-scale, finely annotated wildlife datasets; consequently, its performance may degrade significantly when encountering unseen species or rare animal categories. To address this limitation, future work will explore few-shot learning (e.g., prototype-based meta-learning using only 1–5 support samples per class) and open-set recognition (e.g., energy-based uncertainty calibration or background-aware margin loss) to enable rapid and robust adaptation to novel classes with minimal labeled data. Additionally, to facilitate deployment on resource-constrained UAV platforms such as NVIDIA Jetson, we will investigate a model compression strategy that combines MLKSA attention-guided channel pruning with quantization-aware training (QAT) to further improve computational efficiency and scalability in large-scale ecological monitoring applications.

## Figures and Tables

**Table 1:** Comparison of YOLO-WL and representative detectors on key aspects.

Aspect	YOLOv5/v8	DETR/RT-DETR	YOLO-WL (Ours)
Context Modeling Strategy	Local receptive field	Global self-attention	MSDDSC module
Multi-Scale Capability	Limited	Strong	Explicit multi-scale large-kernel parallelism
Small-Object Adaptivity	Weak	Moderate	Strong (SSA-PAN optimized for small objects)
Attention Mechanism Type	None or simple visual attention	Multi-head self-attention	MLKSA attention
Edge Deployment Friendliness	High	Low	High

**Table 2:** Details of the proposed YOLO-WL network. Layers denotes the module index, From indicates the input source index, and N specifies the number of times the module is stacked.

Components	Layers	From	N	Params	Module	Arguments
Backbone	0	−1	1	464	GCBS	[3, 16, 3, 2]
	1	−1	1	352	GCBS	[16, 32, 3, 2]
	2	−1	1	11,882	C2f-MSDDSC	[32, 32, 1]
	3	−1	1	704	GCBS	[32, 64, 3, 2]
	4	−1	2	82,916	C2f-MSDDSC	[64, 64, 2]
	5	−1	1	1048	GCBS	[64, 128, 3, 2]
	6	−1	2	325,380	C2f-MSDDSC	[128, 128, 2]
	7	−1	1	1408	GCBS	[128, 128, 3, 2]
	8	−1	1	179,330	C2f-MSDDSC	[128, 128, 1]
	9	−1	1	41,344	SPFF	[128, 128, 5]
Neck	10	−1	1	0	Upsample	[None, 2, nearest]
	11	[−1, 6]	1	102	SGF-2	[1]
	12	−1	1	131,840	C2f	[256, 128, 1]
	13	−1	1	0	Upsample	[None, 2, nearest]
	14	[−1, 4]	1	102	SGF-2	[1]
	15	−1	1	37,248	C2f	[192, 64, 1]
	16	−1	1	0	Upsample	[None, 2, nearest]
	17	[−1, 2]	1	102	SGF-2	[1]
	18	−1	1	9408	C2f	[96, 32, 1]
	19	−1	1	990	MLKSA	[32]
	20	−1	1	9280	Conv	[32, 32, 3, 2]
	21	[−1, 4, 15]	1	103	SGF-3	[1]
	22	−1	1	35,200	C2f	[160, 64, 1]
	23	−1	1	990	MLKSA	[64]
	24	−1	1	36,992	Conv	[64, 64, 3, 2]
	25	[−1, 6, 12]	1	103	SGF-3	[1]
	26	−1	1	140,032	C2f	[320, 128, 1]
	27	−1	1	990	MLKSA	[128]
Head	28	[19, 23, 27]	1	346,018	Detect	[6, [32, 64, 128]]
summary: 362 layers, 1,394,688 parameters, 1,394,672 gradients, 9.9 GFLOPs						

**Table 3:** Category statistics of the WAID dataset.

Types	Label	Training Set	Validation Set	Testing Set	Total
Sheep	0	91,495	26,062	13,322	130,879
Cattle	1	44,244	12,604	6239	63,087
Seal	2	15,761	4925	2688	23,374
Camelus	3	4675	1270	675	6620
Kiang	4	3311	836	459	4606
Zebra	5	3791	1000	431	5222

**Table 4:** Category statistics of the Aerial Sheep dataset.

Types	Label	Training Set	Validation Set	Testing Set	Total
Sheep	0	113,541	11,336	5602	129,479

**Table 5:** Category statistics of the AI-TOD dataset.

Types	Label	Training Set	Validation Set	Testing Set	Total
Airplane	0	622	169	744	1535
Bridge	1	511	139	688	1338
Storage-tank	2	5268	2476	5859	13,603
Ship	3	13,538	3790	17,632	34,960
Swimming-pool	4	292	33	291	616
Vehicle	5	248,041	59,903	306,664	614,608
Person	6	14,125	3840	15,442	33,407
Wind-mill	7	175	66	289	530

**Table 6:** Environment configuration.

Environment Item	Configuration Parameters
Operating System	CentOS 8.5.2
GPU	NVIDIA Tesla V100 32 G
CPU	Intel(R) Xeon(R) Platinum 8255C
CUDA	11.2
Memory	314 G
Programming Language	Python3.9
Deep Learning Framework	PyTorch1.8

**Table 7:** Training parameters.

Hyperparameter	Value
Image size	640 × 640
Batch size	64
Epoch	200
Learning rate	0.01
Optimizer	SGD
Momentum	0.937
Weight decay	0.0005

**Table 8:** Performance and complexity of different attention modules.

Model	Precision (%)	Recall (%)	mAP@0.5 (%)	mAP@0.5:0.95 (%)	Params ($10^{6}$)	GFLOPs	Size (MB)
YOLOv8n	91.8	88.0	92.6	56.9	3.0	8.2	6.1
+SEv1	91.1	86.4	92.2	56.1	3.0	8.2	6.2
+SEv2	90.9	88.5	92.8	56.9	3.0	8.2	6.2
+ECA	90.9	88.1	92.5	56.6	3.0	8.2	6.1
+CA	91.0	88.3	92.7	56.4	3.0	8.2	6.2
+SimAM [52]	91.6	88.0	92.8	56.8	3.0	8.2	6.1
+CBAM	91.9	87.4	92.6	56.7	3.1	8.3	6.3
+MLKSA	91.7	88.6	93.2	57.4	3.0	8.2	6.2

**Table 9:** Comparison experiment of shallow feature layer fusion methods.

Model	Precision (%)	Recall (%)	mAP@0.5 (%)	mAP@0.5:0.95 (%)	Params ($10^{6}$)	GFLOPs	Size (MB)
PAN-P3+P4+P5	91.8	88.0	92.6	56.9	3.0	8.2	6.1
PAN-P2+P3+P4+P5	92.7	90.2	94.7	59.2	2.9	12.4	6.2
PAN-P2+P3+P4	93.2	90.2	95.0	59.7	2.0	11.6	4.4
SSA-PAN-P2+P3+P4	93.3	90.4	95.2	59.8	2.0	11.8	4.5

**Table 10:** Ablation results of different modules.

Model	C2f-MSDDSC	MLKSA	SSA-PAN	LFC&GConv	mAP@0.5 (%)	mAP@0.5:0.95 (%)	Params ($10^{6}$)	FLOPs (G)	Size (MB)
Baseline	×	×	×	×	92.6	56.9	3.0	8.2	6.1
Model1	✓	×	×	×	93.2	56.9	3.4	7.5	7.0
Model2	×	✓	×	×	93.2	57.4	3.0	8.2	6.2
Model3	×	×	✓	×	95.2	59.8	2.0	11.8	4.5
Model4	✓	✓	×	×	93.9	58.4	3.4	7.5	7.1
Model5	×	✓	✓	×	95.4	60.0	2.0	11.8	4.5
Model6	✓	×	✓	×	96.1	60.9	2.5	11.1	5.3
YOLO-WL	✓	✓	✓	✓	94.2	58.0	1.4	9.7	3.3

**Table 11:** Comparison of metrics between YOLOv8n and YOLO-WL across categories.

Model	Metrics/%	Sheep	Cattle	Seal	Camelus	Kiang	Zebra	Mean
Baseline	Precision	96.0	95.8	94.4	89.6	82.7	92.3	91.8
	Recall	95.3	91.6	95.9	78.7	80.4	86.0	88.0
	mAP@0.5	97.5	96.3	98.0	88.0	83.1	92.9	92.6
	mAP@0.5:0.95	62.0	64.0	69.5	47.3	43.5	55.1	56.9
YOLO-WL	Precision	96.5	96.8	94.4	90.6	87.2	92.4	93.0
		(+0.5)	(+1.0)	(+0.0)	(+1.0)	(+4.5)	(+0.1)	(+1.2)
	Recall	96.0	90.9	95.1	80.9	78.3	88.9	88.4
		(+0.7)	(−0.7)	(−0.8)	(+2.2)	(−2.1)	(+2.9)	(+0.4)
	mAP@0.5	98.1	97.1	97.7	90.7	86.3	95.1	94.2
		(+0.6)	(+0.8)	(−0.3)	(+2.7)	(+3.2)	(+2.2)	(+1.6)
	mAP@0.5:0.95	62.4	64.5	69.0	49.6	45.1	57.2	58.0
		(+0.4)	(+0.5)	(−0.5)	(+2.3)	(+1.6)	(+2.1)	(+1.1)

**Table 12:** Performance metrics of YOLO-WL under different training set ratios.

Training Set Ratio (%)	Precision (%)	Recall (%)	mAP@0.5 (%)	mAP@0.5:0.95 (%)
20	78.9	67.6	75.6	41.9
40	86.9	83.8	88.5	52.4
60	90.3	86.0	91.5	54.9
80	90.9	87.3	92.6	56.0
100	93.0	88.4	94.2	58.0

**Table 13:** Comparison results of different algorithms on the WAID dataset.

Model	Precision (%)	Recall (%)	mAP@0.5 (%)	mAP@0.5:0.95 (%)	Params ($10^{6}$)	GFLOPs	Size (MB)
YOLOv3 [53]	90.3	77.9	85.4	49.2	12.1	19.0	23.8
YOLOv5n [54]	89.7	87.4	92.0	55.4	2.5	7.2	5.2
YOLOv6n [55]	90.2	85.2	90.8	54.8	4.2	11.9	8.5
YOLOv7t [56]	91.3	89.2	93.2	56.3	6.0	13.2	12.0
YOLOv8n [57]	91.6	88.0	92.5	56.8	3.0	8.2	6.1
YOLOv9t [58]	92.1	88.4	93.1	57.8	2.0	7.9	4.6
YOLOv10n [59]	90.0	87.4	92.9	56.7	2.7	8.4	5.6
YOLOv11n [60]	91.0	87.7	92.8	56.9	2.6	6.4	5.4
YOLOv12n [61]	90.7	85.5	90.9	55.7	2.6	6.5	5.4
YOLO-WL	93.0	88.4	94.2	58.0	1.4	9.7	3.3

**Table 14:** Comparison results of different algorithms on the Aerial Sheep dataset.

Model	Precision (%)	Recall (%)	mAP@0.5 (%)	mAP@0.5:0.95 (%)	Params ($10^{6}$)	GFLOPs	Size (MB)
YOLOv3t [53]	93.8	85.6	92.2	54.6	12.1	19.0	23.8
YOLOv5n [54]	97.6	95.4	97.5	61.1	2.5	7.2	5.2
YOLOv6n [55]	97.8	94.5	97.4	60.6	4.2	11.9	8.5
YOLOv7t [56]	96.1	93.0	96.2	51.6	6.0	13.2	12.0
YOLOv8n [57]	97.8	95.1	97.5	61.5	3.0	8.2	6.1
YOLOv9t [58]	97.9	95.2	97.6	61.6	2.0	7.8	4.5
YOLOv10n [59]	97.7	94.3	97.6	61.0	2.7	8.4	5.6
YOLOv11n [60]	97.4	95.0	97.5	60.8	2.6	6.4	5.4
YOLOv12n [61]	97.7	94.9	97.5	61.3	2.6	6.5	5.4
YOLO-WL	97.5	95.7	98.1	61.7	1.4	9.7	3.3

**Table 15:** Comparison results of different algorithms on the AI-TOD dataset.

Model	Precision (%)	Recall (%)	mAP@0.5 (%)	mAP@0.5:0.95 (%)	Params ($10^{6}$)	GFLOPs	Size (MB)
YOLOv3t [53]	38.7	19.3	20.3	8.4	12.1	19.0	23.8
YOLOv5n [54]	54.5	28.0	29.1	12.3	2.5	7.2	5.2
YOLOv6n [55]	61.0	25.0	25.9	11.1	4.2	11.9	8.5
YOLOv7t [56]	74.0	29.1	30.0	11.9	6.0	13.2	12.0
YOLOv8n [57]	70.7	28.8	30.4	13.0	3.0	8.2	6.1
YOLOv9t [58]	62.7	26.5	29.4	12.5	2.0	7.9	4.5
YOLOv10n [59]	66.0	28.3	29.0	12.6	2.7	8.4	5.6
YOLOv11n [60]	55.5	27.6	29.2	12.4	2.6	6.4	5.4
YOLOv12n [61]	21.9	6.4	12.7	5.2	2.6	6.5	5.4
YOLO-WL	68.7	30.4	32.3	14.0	1.4	9.7	3.3

## Data Availability

The original contributions presented in this study are included in the article. Further inquiries can be directed to the corresponding author(s).

## References

[1] Hughes L.J., Morton O., Scheffers B.R., Edwards D.P. (2023). The ecological drivers and consequences of wildlife trade. Biol. Rev..

[2] Chaves Ó.M., Souza J.C., Buss G., Hirano Z.M.B., Jardim M.M.A., Amaral E.L.S., Godoy J.C., Peruchi A.R., Michel T., Bicca-Marques J.C. (2022). Wildlife is imperiled in peri-urban landscapes: Threats to arboreal mammals. Sci. Total Environ..

[3] Navarro A., Young M., Allan B., Carnell P., Macreadie P., Ierodiaconou D. (2020). The application of Unmanned Aerial Vehicles (UAVs) to estimate above-ground biomass of mangrove ecosystems. Remote Sens. Environ..

[4] Butilă E.V., Boboc R.G. (2022). Urban traffic monitoring and analysis using unmanned aerial vehicles (UAVs): A systematic literature review. Remote Sens..

[5] Lyu X., Li X., Dang D., Dou H., Wang K., Lou A. (2022). Unmanned Aerial Vehicle (UAV) Remote Sensing in Grassland Ecosystem Monitoring: A Systematic Review. Remote Sens..

[6] Patel A., Cheung L., Khatod N., Matijosaitiene I., Arteaga A., Gilkey J.W. (2020). Revealing the Unknown: Real-Time Recognition of Galápagos Snake Species Using Deep Learning. Animals.

[7] Guo X., Shao Q., Li Y., Wang Y., Wang D., Liu J., Fan J., Yang F. (2018). Application of UAV remote sensing for a population census of large wild herbivores—Taking the headwater region of the Yellow River as an example. Remote Sens..

[8] Zhang X., Xuan C., Xue J., Chen B., Ma Y. (2023). LSR-YOLO: A High-Precision, Lightweight Model for Sheep Face Recognition on the Mobile End. Animals.

[9] Roy A.M., Bhaduri J., Kumar T., Raj K. (2023). WilDect-YOLO: An efficient and robust computer vision-based accurate object localization model for automated endangered wildlife detection. Ecol. Inform..

[10] Chen L., Li G., Zhang S., Mao W., Zhang M. (2024). YOLO-SAG: An improved wildlife object detection algorithm based on YOLOv8n. Ecol. Inform..

[11] He A., Li X., Wu X., Su C., Chen J., Xu S., Guo X. (2024). ALSS-YOLO: An Adaptive Lightweight Channel Split and Shuffling Network for TIR Wildlife Detection in UAV Imagery. IEEE J. Sel. Top. Appl. Earth Obs. Remote Sens..

[12] Ma Z., Dong Y., Xia Y., Xu D., Xu F., Chen F. (2024). Wildlife real-time detection in complex forest scenes based on YOLOv5s deep learning network. Remote Sens..

[13] Zhang Y., Cai Z. (2023). CE-RetinaNet: A channel enhancement method for infrared wildlife detection in UAV images. IEEE Trans. Geosci. Remote Sens..

[14] Ye Q., Ma M., Zhao X., Duan B., Wang L., Ma D. (2025). ADD-YOLO: An algorithm for detecting animals in outdoor environments based on unmanned aerial imagery. Measurement.

[15] Jia P., Zhang Y. (2025). A Lightweight Algorithm for Wildlife Detection in Outdoor Environments Based on You Only Look Once Version 8 Network. Eng. Appl. Artif. Intell..

[16] Yuan L., Zhao L., Lai J., Jiang Y., Zhang Q., Shen Z., Zheng C.-H., Huang D.-S. (2024). Icrbp-Lkha: Large Convolutional Kernel and Hybrid Channel-Spatial Attention for Identifying Circrna-Rbp Interaction Sites. PLoS Comput. Biol..

[17] Guo A., Jia Z., Ge B., Chen W., Song S., He C., Zhou G., Wang J., Lv X. (2025). RLCFE-Net: A Reparameterization Large Convolutional Kernel Feature Extraction Network for Weed Detection in Multiple Scenarios. Expert Syst. Appl..

[18] Huang J., Yuan X., Lam C.T., Ke W., Huang G. (2024). Large Kernel Convolution Application for Land Cover Change Detection of Remote Sensing Images. Int. J. Appl. Earth Obs. Geoinf..

[19] Yu F., Koltun V., Funkhouser T. Dilated Residual Networks. Proceedings of the 2017 IEEE Conference on Computer Vision and Pattern Recognition (CVPR).

[20] Yu F., Koltun V. (2015). Multi-scale context aggregation by dilated convolutions. arXiv.

[21] Liu X., Ng A.H.-M., Ge L., Lei F., Liao X. (2024). Multi-branch fusion: A multi-branch attention framework by combining graph convolutional network and CNN for hyperspectral image classification. IEEE Trans. Geosci. Remote Sens..

[22] Yu S., Wang Z., Wang F., Chen K., Yao D., Xu P., Zhang Y., Wang H., Zhang T. (2024). Multiclass classification of motor imagery tasks based on multi-branch convolutional neural network and temporal convolutional network model. Cereb. Cortex.

[23] Lei H., Liu S., Elazab A., Gong X., Lei B. (2020). Attention-guided multi-branch convolutional neural network for mitosis detection from histopathological images. IEEE J. Biomed. Health Inform..

[24] Chen L.-C., Papandreou G., Schroff F., Adam H. (2017). Rethinking atrous convolution for semantic image segmentation. arXiv.

[25] Sun X., Zhang Y., Chen C., Xie S., Dong J. (2023). High-order paired-ASPP for deep semantic segmentation networks. Inf. Sci..

[26] Lin T.-Y., Dollar P., Girshick R., He K., Hariharan B., Belongie S. Feature pyramid networks for object detection. Proceedings of the IEEE Conference on Computer Vision and Pattern Recognition (CVPR).

[27] Kim S.W., Kook H.K., Sun J.Y., Kang M.C., Ko S.J. Parallel feature pyramid network for object detection. Proceedings of the European Conference on Computer Vision (ECCV).

[28] Liu S., Qi L., Qin H., Shi J., Jia J. Path aggregation network for instance segmentation. Proceedings of the IEEE Conference on Computer Vision and Pattern Recognition (CVPR).

[29] Tan M., Pang R., Le Q.V. Efficientdet: Scalable and efficient object detection. Proceedings of the IEEE/CVF Conference on Computer Vision and Pattern Recognition (CVPR).

[30] Ghiasi G., Lin T.Y., Le Q.V. Nas-fpn: Learning scalable feature pyramid architecture for object detection. Proceedings of the 2019 IEEE/CVF Conference on Computer Vision and Pattern Recognition (CVPR).

[31] Han K., Wang Y., Chen H., Chen X., Guo J., Liu Z., Tang Y., Xiao A., Xu C., Xu Y. (2023). A Survey on Vision Transformer. IEEE Trans. Pattern Anal. Mach. Intell..

[32] Hassanin M., Anwar S., Radwan I., Khan F.S., Mian A. (2024). Visual Attention Methods in Deep Learning: An In-Depth Survey. Inf. Fusion.

[33] Selvaraju R.R., Cogswell M., Das A., Vedantam R., Parikh D., Batra D. (2019). Grad-CAM: Visual Explanations from Deep Networks Via Gradient-Based Localization. Int. J. Comput. Vis..

[34] Wang H., Wang Z., Du M., Yang F., Zhang Z., Ding S., Mardziel P., Hu X. Score-CAM: Score-Weighted Visual Explanations for Convolutional Neural Networks. Proceedings of the IEEE/CVF Conference on Computer Vision and Pattern Recognition (CVPR) Workshops.

[35] Hu J., Shen L., Sun G. Squeeze-and-excitation networks. Proceedings of the 2018 IEEE/CVF Conference on Computer Vision and Pattern Recognition.

[36] Narayanan M. (2023). SENetV2: Aggregated dense layer for channelwise and global representations. arXiv.

[37] Wang Q., Wu B., Zhu P., Li P., Zuo W., Hu Q. ECA-Net: Efficient channel attention for deep convolutional neural networks. Proceedings of the IEEE/CVF Conference on Computer Vision and Pattern Recognition (CVPR).

[38] Zhang H., Dana K., Shi J., Zhang Z., Wang X., Tyagi A., Agrawal A. Context encoding for semantic segmentation. Proceedings of the IEEE Conference on Computer Vision and Pattern Recognition.

[39] Jaderberg M., Simonyan K., Zisserman A. (2015). Spatial transformer networks. Adv. Neural Inf. Process. Syst..

[40] Almahairi A., Ballas N., Cooijmans T., Zheng Y., Larochelle H., Courville A. Dynamic capacity networks. Proceedings of the 33rd International Conference on Machine Learning, PMLR.

[41] Woo S., Park J., Lee J.Y., Kweon I.S. Cbam: Convolutional block attention module. Proceedings of the European Conference on Computer Vision (ECCV).

[42] Park J., Woo S., Lee J.Y., Kweon I.S. (2018). Bam: Bottleneck attention module. arXiv.

[43] Hou Q., Zhou D., Feng J. Coordinate attention for efficient mobile network design. Proceedings of the IEEE/CVF Conference on Computer Vision and Pattern Recognition (CVPR).

[44] Dosovitskiy A., Beyer L., Kolesnikov A., Weissenborn D., Zhai X., Unterthiner T., Dehghani M., Minderer M., Heigold G., Gelly S. (2020). An image is worth 16x16 words: Transformers for image recognition at scale. arXiv.

[45] Liu Z., Lin Y., Cao Y., Hu H., Wei Y., Zhang Z., Lin S., Guo B. Swin transformer: Hierarchical vision transformer using shifted windows. Proceedings of the IEEE/CVF International Conference on Computer Vision (ICCV).

[46] Carion N., Massa F., Synnaeve G., Usunier N., Kirillov A., Zagoruyko S. (2020). End-to-end object detection with transformers. European Conference on Computer Vision.

[47] Zhu X., Su W., Lu L., Li B., Wang X., Dai J. (2020). Deformable detr: Deformable transformers for end-to-end object detection. arXiv.

[48] Zhang H., Li F., Liu S., Zhang L., Su H., Zhu J., Ni L.M., Shum H.-Y. (2022). Dino: Detr with improved denoising anchor boxes for end-to-end object detection. arXiv.

[49] Zhao Y., Lv W., Xu S., Wei J., Wang G., Dang Q., Liu Y., Chen J. Detrs beat yolos on real-time object detection. Proceedings of the IEEE/CVF Conference on Computer Vision and Pattern Recognition (CVPR).

[50] Mou C., Liu T., Zhu C., Cui X. (2023). Waid: A large-scale dataset for wildlife detection with drones. Appl. Sci..

[51] Wang J., Yang W., Guo H., Zhang R., Xia G.S. Tiny object detection in aerial images. Proceedings of the 25th International Conference on Pattern Recognition (ICPR).

[52] Yang L., Zhang R.-Y., Li L., Xie X. Simam: A simple, parameter-free attention module for convolutional neural networks. Proceedings of the 38th International Conference on Machine Learning, PMLR.

[53] Redmon J., Farhadi A. (2018). Yolov3: An incremental improvement. arXiv.

[54] Ultralytics (2020). YOLOv5. https://github.com/ultralytics/yolov5.

[55] Li C., Li L., Jiang H., Weng K., Geng Y., Li L., Ke Z., Li Q., Cheng M., Nie W. (2022). YOLOv6: A Single-Stage Object Detection Framework for Industrial Applications. arXiv.

[56] Wang C.Y., Bochkovskiy A., Liao H.Y.M. YOLOv7: Trainable bag-of-freebies sets new state-of-the-art for real-time object detectors. Proceedings of the IEEE/CVF Conference on Computer Vision and Pattern Recognition (CVPR).

[57] Ultralytics (2023). YOLOv8. https://github.com/ultralytics/ultralytics.

[58] Wang C.Y., Yeh I.H., Liao H.Y.M. (2025). YOLOv9: Learning What You Want to Learn Using Programmable Gradient Information. European Conference on Computer Vision (ECCV).

[59] Wang A., Chen H., Liu L., Chen K., Lin Z., Han J., Ding G. (2024). YOLOv10: Real-Time End-to-End Object Detection. arXiv.

[60] Ultralytics (2024). YOLOv11. https://github.com/ultralytics/ultralytics.

[61] Tian Y., Ye Q., Doermann D. (2025). YOLOv12: Attention-Centric Real-Time Object Detectors. arXiv.

